# TiO_2_-graphene oxide nanocomposite as advanced photocatalytic materials

**DOI:** 10.1186/1752-153X-7-41

**Published:** 2013-02-27

**Authors:** Václav Štengl, Snejana Bakardjieva, Tomáš Matys Grygar, Jana Bludská, Martin Kormunda

**Affiliations:** 1 Department of Solid State Chemistry, Institute of Inorganic Chemistry AS CR v.v.i., 250 68, Řež, Czech Republic; 2 Department of Physics, Faculty of Science, J.E.Purkyně University in Ústí nad Labem, 400 96, Ustí n. L, Czech Republic

**Keywords:** Graphene, Titanium(IV) oxide, Graphene oxide, Photocatalysis

## Abstract

**Background:**

Graphene oxide composites with photocatalysts may exhibit better properties than pure photocatalysts via improvement of their textural and electronic properties.

**Results:**

TiO_2_-Graphene Oxide (TiO_2_ - GO) nanocomposite was prepared by thermal hydrolysis of suspension with graphene oxide (GO) nanosheets and titania peroxo-complex. The characterization of graphene oxide nanosheets was provided by using an atomic force microscope and Raman spectroscopy. The prepared nanocomposites samples were characterized by Brunauer–Emmett–Teller surface area and Barrett–Joiner–Halenda porosity, X-ray Diffraction, Infrared Spectroscopy, Raman Spectroscopy and Transmission Electron Microscopy. UV/VIS diffuse reflectance spectroscopy was employed to estimate band-gap energies. From the TiO_2_ - GO samples, a 300 μm thin layer on a piece of glass 10×15 cm was created. The photocatalytic activity of the prepared layers was assessed from the kinetics of the photocatalytic degradation of butane in the gas phase.

**Conclusions:**

The best photocatalytic activity under UV was observed for sample denoted TiGO_100 (k = 0.03012 h^-1^), while sample labeled TiGO_075 (k = 0.00774 h^-1^) demonstrated the best activity under visible light.

## Introduction

Traditionally, TiO_2_ is currently known as the most available and commercially cheapest photocatalyst. It has been well documented that GO is heavily oxygenated graphene that is readily exfoliated in water to yield stable dispersions consisting mostly of single-layer sheets. The use of graphene oxide as the nanoscale substrates for the formation of nonocomposites with metal oxides is widely explored due to an idea to obtain a hybrid which could be combined both properties of GO as fascinating paper-shape material and the features of single nano-sized metal oxide particles.

Recent studies concentrate on the preparation and applications of GO modified photocatalyst because their large specific surface area and high activity in most catalytic processes. Krishnamoorthy et al. [1] presented an article where photocatalytic characteristics and properties of graphene oxide were investigated by measuring the reduction rate of resazurin into resorufin as a function of UV irradiation time. The synthesis and physiochemical characterization of titanium oxide nanoparticle-graphene oxide (TiO_2_-GO) and titanium oxide nanoparticle-reduced graphene oxide (TiO_2_-RGO) composites were early provided and emphasized on the different synthetic strategies for the preparation of graphene-TiO_2_ materials. For example, TiO_2_-GO materials have been prepared via the hydrolysis of TiF_4_ at 60°C for 24 h in the presence of an aqueous dispersion of graphene oxide (GO). The reaction proceeded to yield an insoluble material that is composed of TiO_2_ and GO [2].

In an another strategy graphene oxide/TiO_2_ composites were prepared by using TiCl_3_ and graphene oxide as reactants. The concentration of graphene oxide in starting solution played an important role in photoelectronic and photocatalytic performance of graphene oxide/TiO_2_ composites. In this way either a p-type or n-type semiconductive graphene oxide/TiO_2_ composites were synthesized. These semiconductors were excited by visible light with wavelengths longer than 510 nm and acted as sensitizers in graphene TiO_2_ composites [3].

TiO_2_-Graphene Oxide intercalated composite has been successfully prepared at low temperature (80°C) with graphite oxide and titanium sulfate Ti(SO_4_)_2_ as initial reactants. Graphite oxide was firstly exfoliated by NaOH and formed single and multi-layered graphite oxide mixture which can be defined as graphene oxide, [TiO]^2+^ induced by the hydrolysis of Ti(SO_4_)_2_ diffused into graphene oxide interlayer by electrostatic attraction [4].

Recently, Min et al. [5] presented a novel dye-sensitized photocatalytic system for photochemical energy conversion employing reduced graphene oxide (RGO) as a catalyst scaffold and an efficient electron relay mediator between photosensitizer and catalyst. Under visible light irradiation (λ = 420 nm), the photocatalyst of Eosin Y sensitized RGO with dispersed Pt nanoparticles exhibited highly efficient activity for hydrogen evolution from water reduction.

A new technique for producing of novel solvent-exfoliated graphene-TiO_2_ nanocomposites was published [6], which are then compared to previously reported reduced graphene oxide-TiO2 nanocomposites in an effort to elucidate the role of graphene and its defects in the photocatalytic reduction of CO_2_ to CH_4_. Another novel method [7] was developed to synthesize graphite oxide/TiO_2_ composites as a highly efficient photocatalyst by in situ depositing TiO_2_ nanoparticles on graphene oxide nano-sheets by a liquid phase deposition, followed by a calcination treatment at 200°C.

In our previous work [8], we reported on nonstoichiometric TiO_2_-graphene oxide nanocomposite, which was prepared by thermal hydrolysis of suspension with graphene oxide nanosheets and titania peroxo-complex. It should be mentioned that we are able to produce pure graphene nanosheets in large quantity from natural graphite by using high intensity cavitation field in the high-pressure ultrasonic reactor. Graphene oxide sheets, prepared by such safe and friendly method, can be used as a good support for TiO_2_ to enhance its photocatalytic activity. In this paper, we reported on an "one-pot" thermal hydrolysis of titanium peroxo-complexes in the presence of graphene oxide in aqueous solution. Our "hydrogen peroxide route" is based on hydrolysis of Ti(IV) sulfate, next treatment of the resulting gel by hydrogen peroxide, and thermal decomposition of so formed peroxo-titanate under reflux at ambient pressure producing directly the TiO_2_-GO photocatalyst. The relative photocatalytic activity of the as-prepared thin layers of titania/graphene nanocomposite in poly(hydroxyethyl methacrylate) was assessed by the photocatalytic decomposition of butane under UV and visible light.

### Preparation TiO_2_ - GO nanocomposite

All chemical reagents used in the present experiments were obtained from commercial sources and used without further purification. Titanium oxo-sulfate TiOSO_4_, hydrogen peroxide H_2_O_2_, ethylene glycol OHCH_2_CH_2_OH and ammonium hydroxide NH_4_OH were supplied by Sigma-Aldrich. Graphene was produced in large quantity from natural graphite (Koh-i-noor Grafite Ltd. Czech republic) using high intensity cavitation field in a pressure batch-ultrasonic reactor (UIP 2000hd, 20 kHz, 2000 W, Hielscher Ultrasonics GmbH) [9].

Graphene oxide was prepared by our safety method, a 60 ml of H_2_SO_4_ and 10 ml of H_3_PO_4_, 1 g of graphene and 3 g of KMnO_4_ were mixed in round bottom flask. The reaction was then heated to 40°C and stirred for 6 hours and pink squash suspension was obtained. Subsequently was poured onto ice with 200 ml of 30% H_2_O_2_. The pink squash suspension quickly changed to lemon-like yellow suspension. The whole reaction product was purged by dialysis (Spectra/Por 3 dialysis membrane), washed with ethanol and dried at 105°C.

In the typical procedure of TiO_2_ - GO nanocomposite preparation, 100 ml of 1.6 M titanium oxo-sulfate (TiOSO_4_) was hydrolyzed by slow addition of ammonium hydroxide solution (10%) under constant stirring at temperature of 0°C in ice bath. The stirring last until the reaction mixture reaches pH 8.0. The obtained white precipitate was separated by filtration. The consequent depuration of sulfate ions from precipitate with distilled water was confirmed by the BaCl_2_. The wet precipitate is mixed with 100 ml of 15% hydrogen peroxide solution; thereby a yellow solution of titania peroxo-complex is obtained.

Well defined quantity (see Table 1) of GO nanosheets was dispersed using ultrasound in water, added to the yellow precursor of titania peroxo-complex and annealed at a heated mantle in a round-bottom flask with a reflux cooler at 100°C for 48 hours. The originated blue TiO_2_ - GO nanocomposite was filtered off and dried at 105°C. Ten samples of TiO_2_-GO nanocomposites doped as TiGO_XXX, where XXX are grams of GO, were prepared.

**Table 1:** Sample composition, crystallite size, BET a total pore volume of prepared samples

**Sample**	**Graphene oxide [g]**	**Crystallite size [nm]**	**Cell parameter a [Å]**	**Cell parameter c [Å]**	**BET [m**^**2**^**g**^**-1**^**]**	**Total pore volume [ cm**^**3**^**g**^**-1**^**]**
TiGO_001	0.005	49.6	3.7963(2)	9.5144(5)	100..2	0.6530
TiGO_005	0.001	51.9	3.7968(4)	9.5121(4)	151.4	0.7846
TiGO_010	0.01	52.2	3.7970(2)	9.5121(6)	195.7	1.4668
TiGO_050	0.05	34.5	3.7970(3)	9.5086(8)	185.7	0.623
TiGO_075	0.075	34.5	3.7973(2)	9.5077(7)	119.2	0.6501
TiGO_100	0.100	32.1	3.7973(6)	9.5060(4)	168.4	0.6687
TiGO_200	0.200	35.0	3.7971(4)	9.5047(5)	178.0	0.6247
TiGO_300	0.300	46.7	3.7954(5)	9.5118(7)	78.9	0.618
TiGO_400	0.400	43.6	3.7954(2)	9.5106(6)	68.6	0.5545
TiGO_500	0.500	39.1	3.7955(6)	9.5095(8)	78.1	0.6089

The prepared TiO_2_ - GO nanocomposite powder (2 g) was dispersed in a mixture of 5 ml poly(hydroxyethyl methacrylate) and 10 ml of ethanol. From this suspension, a 300 μm thin layer on a piece of glass, 100 × 150 mm was created.

### Characterisation methods

Diffraction patterns were collected with diffractometer PANalytical X´Pert PRO equipped with conventional X-ray tube (Cu Kα radiation, 40 kV, 30 mA) and a linear position sensitive detector PIXcel with an anti-scatter shield. A programmable divergence slit set to a fixed value of 0.5 deg, Soller slits of 0.02 rad and mask of 15 mm were used in the primary beam. A programmable anti-scatter slit set to fixed value of 0.5 deg., Soller slit of 0.02 rad and Ni beta-filter were used in the diffracted beam. Qualitative analysis was performed with the DiffracPlus Eva software package (Bruker AXS, Germany) using the JCPDS PDF-2 database [10]. For quantitative analysis of XRD patterns we used Diffrac-Plus Topas (Bruker AXS, Germany, version 4.1) with structural models based on ICSD database [11]. This program permits to estimate the weight fractions of crystalline phases and mean coherence length by means of Rietveld refinement procedure.

AFM images were obtained using an NTEGRA Aura (NT-MTD) microscope. A sample of the diluted dispersion was placed on synthetic mica as an atomically smooth support and evaporated at room temperature. The measurements were performed in air at room temperature in non contact mode, with Si tips of the 1650–00 type at resonance frequencies ranging from 180 to 240 kHz.

The morphology of sample powders was inspected by transmission electron microscopy (TEM) and the crystal structure was analyzed by electron diffraction (ED) using a 200 kV TEM microscope JEOL 2010 F. As specimen support for TEM investigations a microscopic copper grid covered by a thin transparent carbon film was used. The samples were studied in both bright field and by electron diffraction with a selecting aperture (SAED) mode at an acceleration voltage of 200 kV.

The surface areas of samples were determined from nitrogen adsorption–desorption isotherms at liquid nitrogen temperature using a Coulter SA3100 instrument with outgas 15 min at 150°C. The Brunauer–Emmett–Teller (BET) method was used for surface area calculation [12], the pore size distribution (pore diameter, pore volume and micropore surface area of the samples) was determined by the Barrett–Joyner–Halenda (BJH) method [13].

The Raman spectra were acquired with DXR Raman microscope (Thermo Scientific) with 532 nm (6 mW) laser, 32 two-second scans were accumulated with laser 532 nm (6 mW) under 10× objective of Olympus microscope.

Infrared spectra were recorded by using Thermo-Nicolet Nexus 670 FT-IR spectrometer approximately in 4000–500 and 500–50 cm^-1^, respectively, with single-reflection horizontal accessory on Si crystal. The samples were mixed with KBr and pressed to conventional pellets at ambient conditions and measured in the transmission mode.

XPS apparatus was equipped with SPECS X-Ray XR50 (Al cathode 1486.6 eV) and SPECS PHOIBOS 100 Hemispheric Analyzer with 5-channels detector. A background pressure in XPS during the measurements was under 2×10^-8^ mbar. XPS survey-scan spectra were made at pass energy of 40 eV; the energy resolution was set to 0.5 eV. While individual high-resolution spectra were taken at pass energy of 10 eV with 0.05 eV energy steps. A software tool CasaXPS was used to fit high-resolution multi components peaks. The proper surface charge compensation was done by fitting C-C, C-H component of C 1 s peak to reference binding energy 284.5 eV. The atomic concentration of compounds was evaluated with relative sensitivity factors (RSF) defined in standard table of CasaXPS software.

Diffuse reflectance UV/VIS spectra for evaluation of photo-physical properties were recorded in the diffuse reflectance mode (R) and transformed to absorption spectra through the Kubelka-Munk function [14]. A Perkin Elmer Lambda 35 spectrometer equipped with a Labsphere RSAPE- 20 integration sphere with BaSO_4_ as a standard was used. The reflectance data were obtained as relative percentage reflectance to a non absorbing material (BaSO_4_) which can optically diffuse light.

Kinetics of the photocatalytic degradation of butane (0.87%) was measured by using a home-made stainless steel batch photo-reactor [15] with a Narva black-light fluorescent lamp at wavelength 365 nm and warm-white fluorescent lamp at wavelength up to 400 nm (input power 8 W, light intensity 6.3 mW cm^-2^). The gas concentration was measured with the use of Quadrupole Mass Spectrometer JEOL JMS-Q100GC and gas chromatograph Agilent 6890 N. A high-resolution gas chromatography column (19091P-QO4, J&W Scientific) was used. Samples were taken from the reactor automatically through the sampling valve (6-port external volume sample injector VICI, Valco Instruments Co. Inc.) in a time interval of 2 hours.

Blank tests (a layer of poly(hydroxyethyl methacrylate) without titania) were performed in order to establish the effect of photolysis and catalysis on the conversion of butane. The UV irradiation detects that there was none or immeasurable conversion of butane, as a testing gas, into CO and/or CO_2_, and consequently neither butane absorbed on the poly(hydroxyethyl methacrylate) matrix. The injection volume of butane into the photoreactor was 30 ml.

## Results and discussion

**Figure 1:**
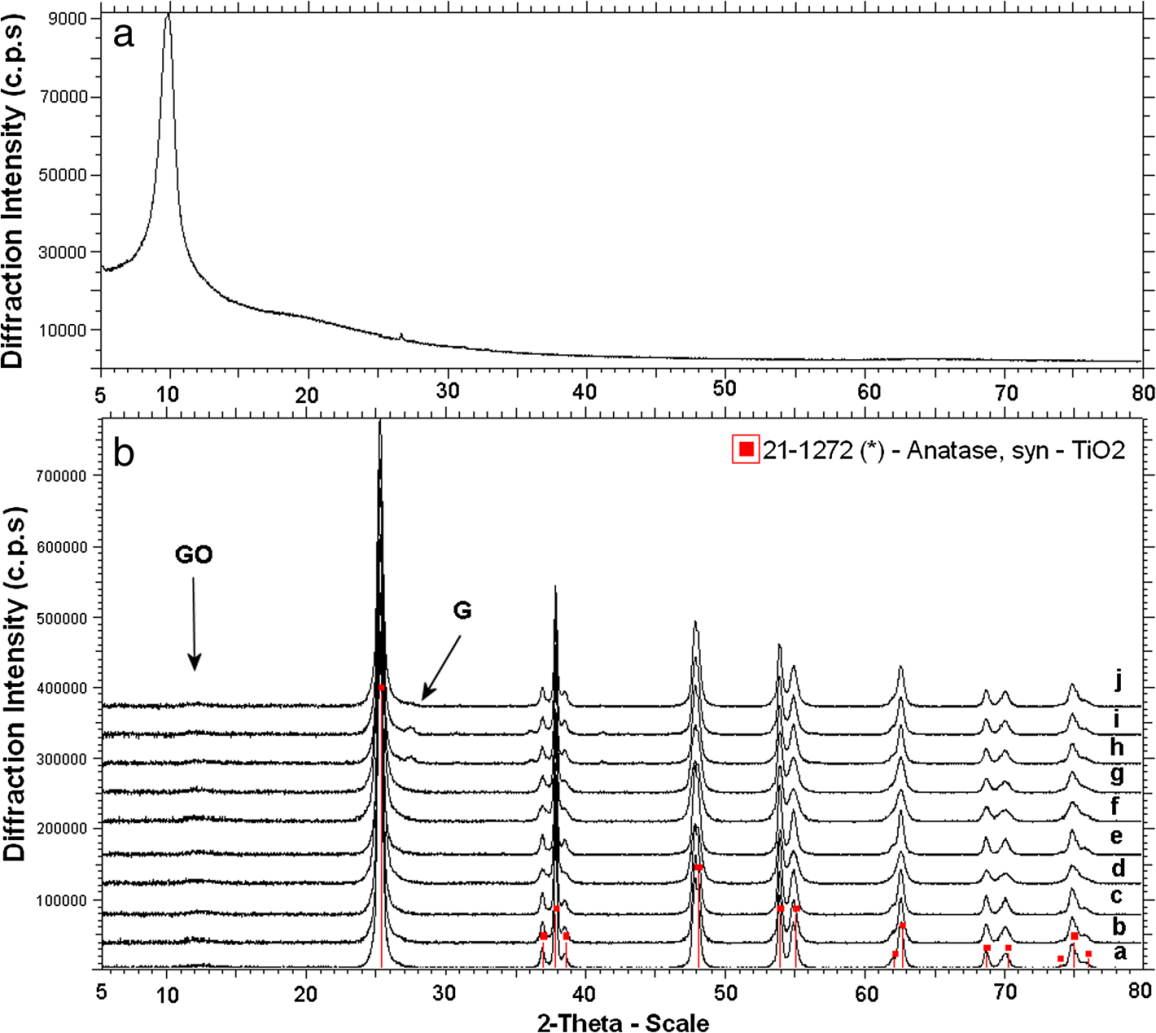
**a The XRD pattern of graphene oxide. b** The XRD patterns of TiO_2_-GO nanocomposites, diffraction line **a**) TiGO_001, **b**) TiGO_005, **c**) TiGO_010, **d**) TiGO_050, **e**) TiGO_075, **f**) TiGO_100, **g**) TiGO_200, **h**) TiGO_300, **i**) TiGO_400 and **j**) TiGO_500.

Early published experimental XRD pattern of graphene oxide demonstrated arising of strong 001 reflection peak at 2θ ~10° with a basal spacing of d_001_ = 6.33 Å [16]. We calculated d-spacing of sample prepared during our experiment (see Figure 1a) such as GO d_001_ = 6.718 Å which is in good agreement with published results [16,17].

The Figure 1b shows X-ray diffraction patterns of TiO_2_-GO nanocomposites. All samples show only the anatase phase (PDF 21–1272). Higher concentrations of GO (samples TiGO_300 and TiGO_400) probably lead to partial reduction of GO to graphene and a weak peak at 2Θ = 26.50° appears (see arrow G). Nevertheless, the intensity of graphene X-ray lines is under possibility for quality Rietveld refinement. Furthermore, in the position at 2Θ ~ 10-12° (see arrow GO), there is an obvious hint of GO peak, which is due to the small particle size and its concentration featureless.

Crystallite size, a and c cell parameters of anatase, calculated by the Rietveld refinement procedure using Topas v.4.2 programme, based on the full width at half-maximum of the peak at 25.4° are presented in Table 1; the crystallite size is in range from ∼ 32 to 52 nm. The cell parameters of pure anatase are a = 3.7845 Å and c = 9.5143 Å; an increasing of lattice parameters a and c for the titania/graphene oxide nanocomposite is expected to occur if some of the Ti^4+^ are transformed to Ti^3+^, because of the larger ionic radius of Ti^3+^ (0.670 Å) compared to Ti^4+^ (0.605 Å) [18].

Atomic force microscopy and electron transmission microscopy were used to determine quality of delamination of graphene oxide (GO) used for preparation TiO_2_-GO nanocomposite.

**Figure 2:**
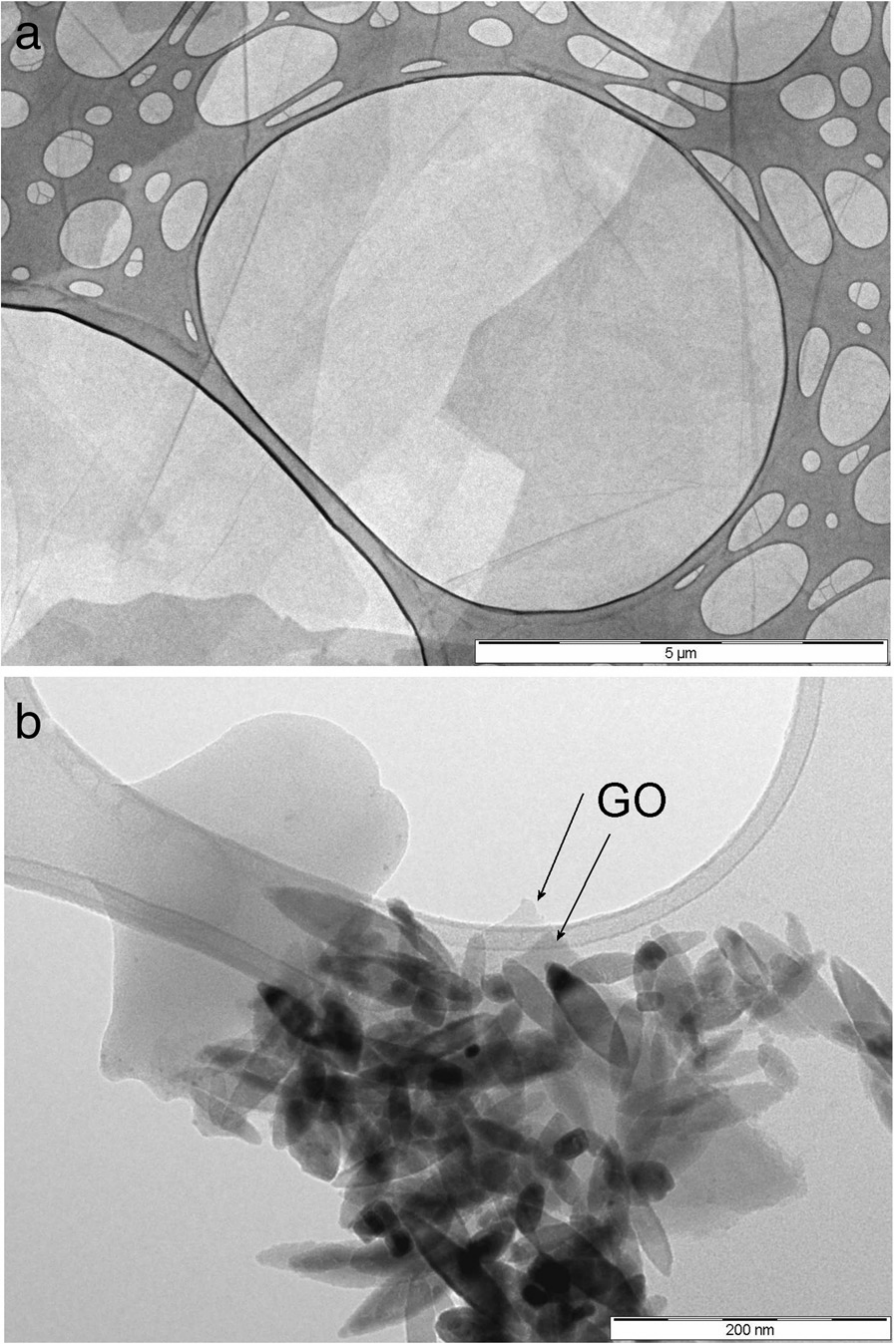
**a) TEM images of graphene oxide (GO), b) TEM images of TiO**_**2**_**-GO nanocomposite.**

In our previously work we have been demonstrated that the thermal hydrolysis of titania peroxo-complex leads to spindle-like particles [19]. Our experiments were confirmed that direct interaction between TiO_2_ nanoparticles and graphene oxide sheet prevents the re-aggregation of the sheets of graphene oxide. The TEM images of GO prepared by modified oxidation method is presented in Figure 2a. It follows from the picture, that GO formed big smooth plates of size ~ 5×5 μm. Figure 2b shows TEM images of TiO_2_-GO nanocomposite and demonstrates that TiO_2_ nanoparticles were dispersed uniformly on the graphene plane.

**Figure 3:**
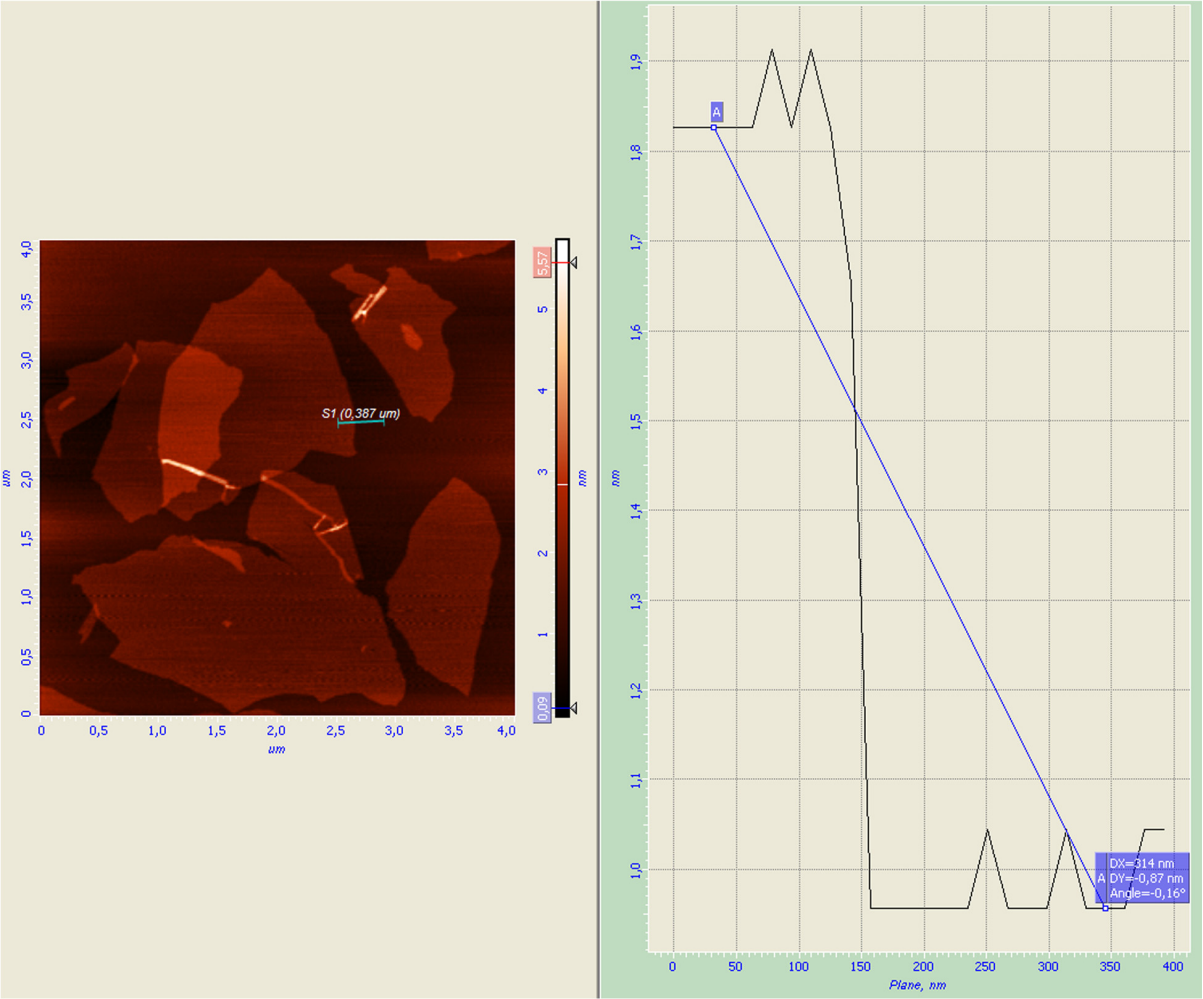
AFM images of prepared graphene oxide.

Figure 3 shows AFM images of GO with a thickness of 0.87 nm according to cross-sectional analysis, which is comparable with the interlayer spacing 0.91 nm of GO measured by X-ray powder diffraction (see Figure 1a). These results indicate that the exfoliation of graphite oxide down to monolayer sheets of GO is successfully obtained [16].

**Figure 4:**
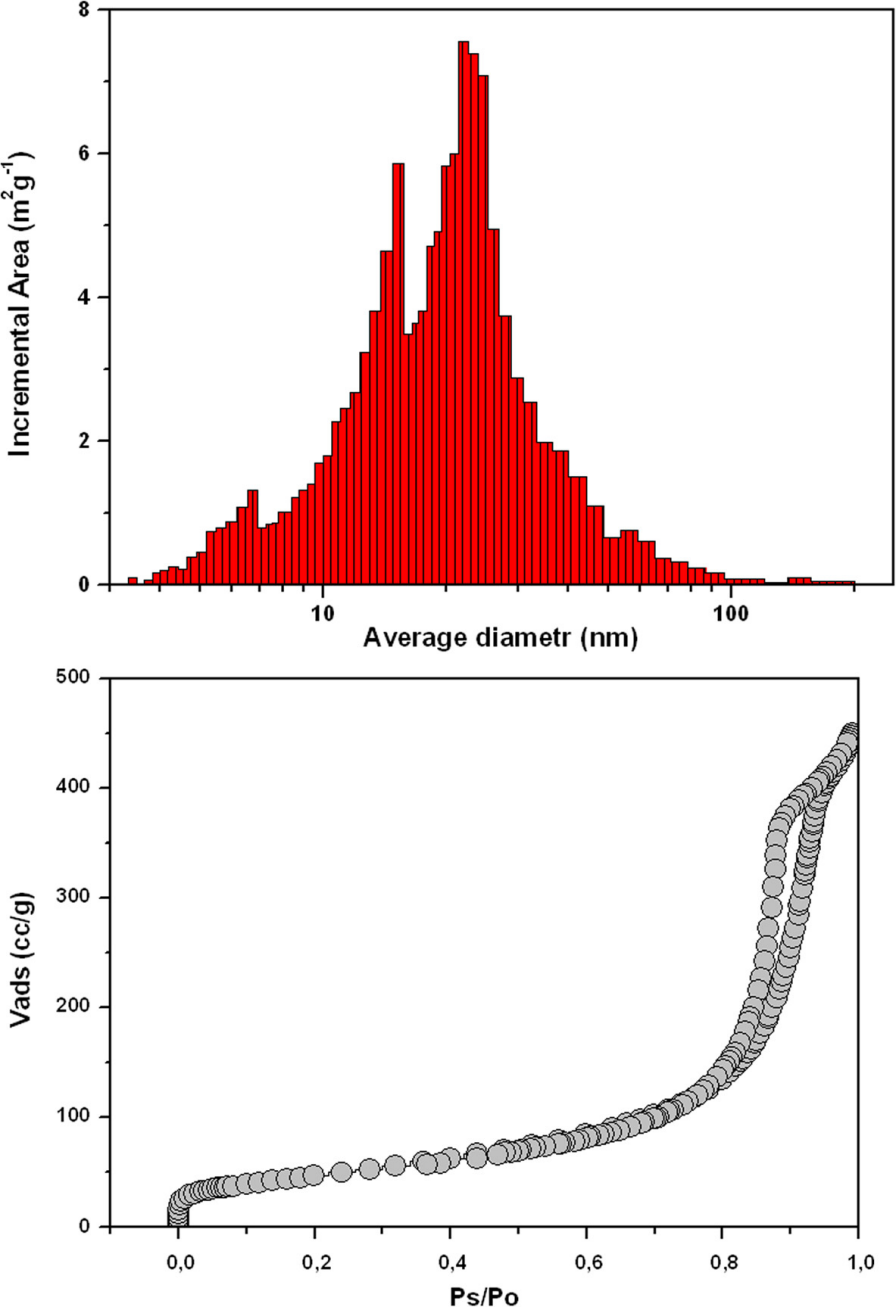
**Dependence of pore area on pore diameter and nitrogen adsorption/desorption isotherms of TiO**_**2**_**-GO nanocomposite.**

The specific surface area of the prepared TiO_2_-GO nanocomposites calculated by the multi-point Brunauer-Emmett-Teller (BET) method and total pore volume are listed in Table 1. A typical Barrett-Joyner-Halenda (BJH) pore-size distribution plot and nitrogen adsorption/desorption isotherms are shown in Figure 4. According to IUPAC notation [20], microporous and macroporous materials have pore diameters smaller than 2 and greater than 50 nm, respectively; the mesoporous category thus lies in the middle. As for all the titania/graphene nanocomposites, the isotherm form corresponds to that of type IV isotherm classification and the type E hysteresis loop is in agreement with the De Boer classification attributed to mesoporous solids [21]. The maximum of average pore size lies between 20 and 35 nm and all prepared TiO_2_-GO nanocomposites have mesoporous texture. With increasing content of GO in the composite decreasing trend of specific surface area is obvious (see Table 1). This may be due to agglomeration GO, which occurs at higher concentrations. Graphene oxide nanosheets are coated with titania nanoparticles. Therefore when the surface area of TiO_2_-GO nanocomposites is measured, its size is dominantly determined by surface properties of anatase nanoparticles. For this reason, relatively low specific surface of the TiO_2_-GO nanocomposites (200 m^2^g^-1^) was found compared to the theoretical value of the graphene and graphene oxide, respectively. Nevertheless mesoporous TiO_2_-graphene oxide nanocomposites have specific surface area in interval ~ 80–200 m^2^g^-1^ being considerably larger than those of P25 and similarly prepared neat TiO_2_ particles without using graphene oxide.

Raman spectroscopy is a powerful tool to characterize the crystalline quality of graphene or GO. Additional file 1: Figure S1 shows the Raman spectrum of GO. In case of thermal hydrolysis of titania peroxo-complexes in the presence of GO there is no reduction of GO. Due to the bluish coloration of samples can be assumed, on the contrary, that there is reduction of Ti^4+^ to Ti^3+^[22]. However, if there is a reduction of Ti^4+^, somewhere an oxidation must occur, and it is probably on the graphene skeleton in free positions of Π bonds. Following oxidation of graphene due to reduction of Ti^4+^ corresponds to an increase of intensity of Raman bands GO in the nano-composite TiO_2_-GO.

In our Raman spectrum, the G band is broadened and shifted slightly to 1605 cm^-1^, whereas the intensity of the D band at 1350 cm^-1^ increases substantially. Increased background of the spectrum is caused by the sample fluorescence. G band is common to all sp2 carbon forms and provides information on the in-plane vibration of sp2 bonded carbon atoms and the D band suggests the presence of sp3 defects [3].

**Figure 5:**
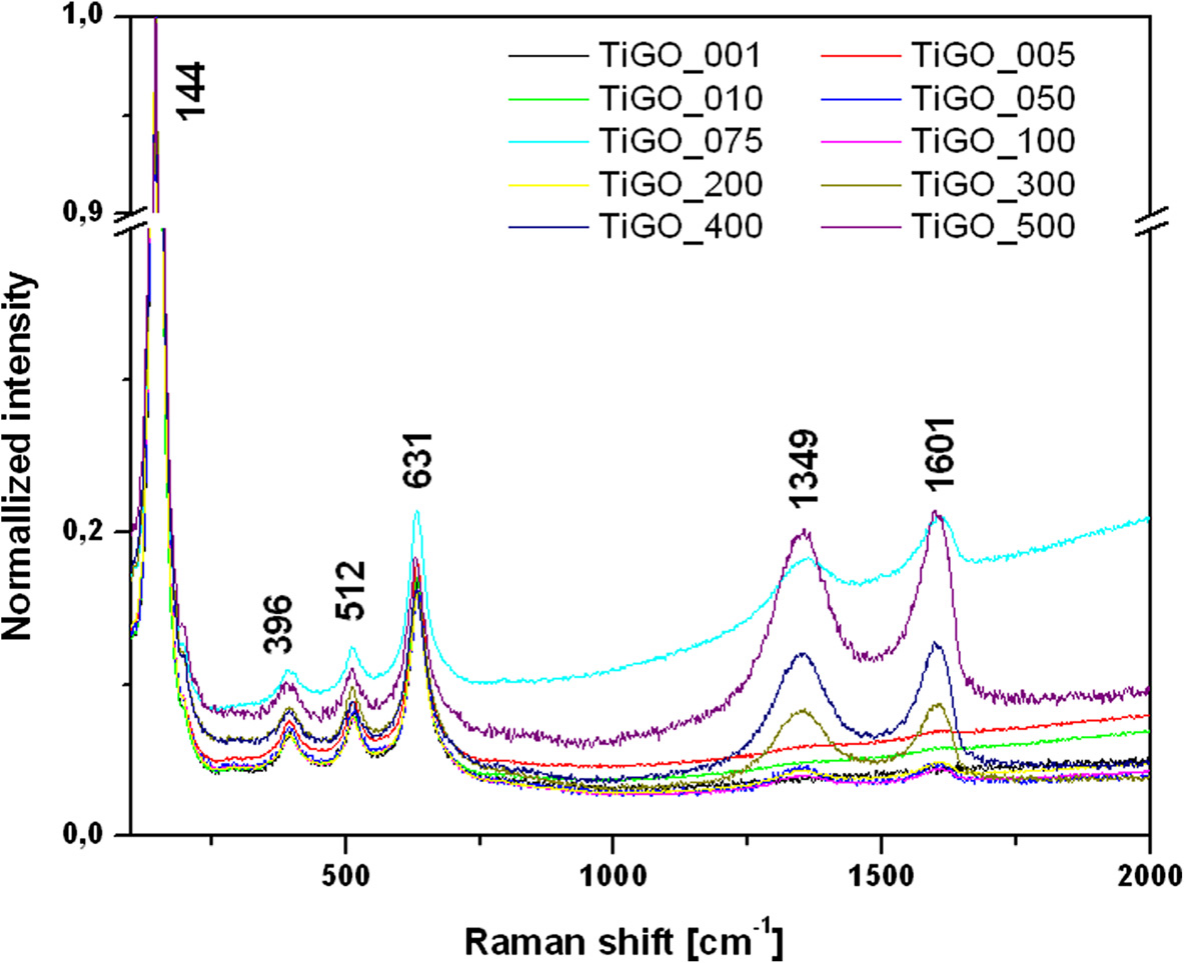
**The Raman spectra of TiO**_**2**_**-GO composites.**

The Raman spectra of series nanocomposites TiO_2_-GO is presented in Figure 5. The specific vibration modes are located at 144 cm^-1^ (Eg), 396 cm^-1^ (B1g), 512 cm^-1^ (B1g + A1g) and 631 cm^-1^ (Eg) indicating the presence of the anatase phase in all of these samples. The D bands of GO are located at 1349 cm^-1^ and G band at 1601 cm^-1^. With increasing concentrations of GO the intensity of both bands also increases. The background of the sample labeled TiGO_075 is increased by the sample fluorescence.

**Figure 6:**
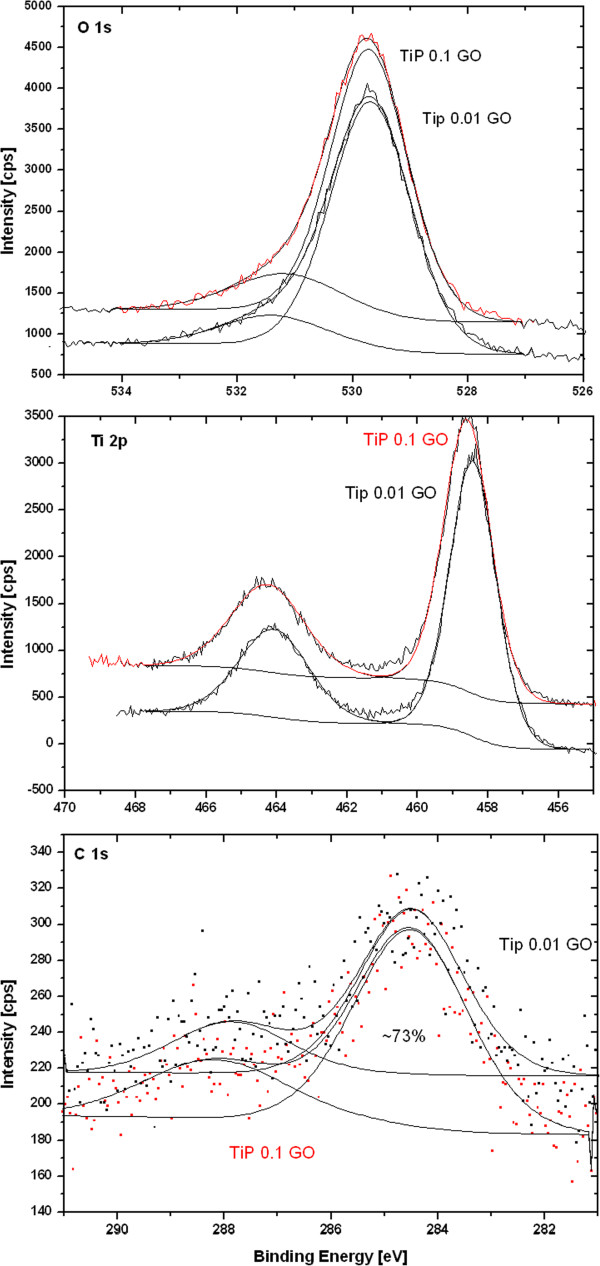
**High resolution XPS spectra of TiO**_**2**_**-GO composites.**

High resolution XPS spectra were accumulated 10 times to enhance the signal and to noise ratio, and are presented in Figure 6. The composition of the TiO_2_-GO nanocomposites was evaluated from the high resolution spectra of O 1 s, C 1 s and Ti 2p. The compositions are summarized in the Additional file 1: Table 1. The O/Ti ratio was evaluated and the results show over stoichiometric values. The high O/Ti ratio can be partially explained by the fact that the O1s spectra show a main peak at 529.7 eV with a shoulder at about ~531.4 eV. The peak at 529.7 eV is assigned to oxygen lattice, while the shoulder may be attributed to oxygen in adsorbed hydroxyl groups.

The binding energy calibration was made on C-C component of C 1 s peak after a deconvolution process. The intensity of C 1 s peak is rather small and the signal is noisy, but the C 1 s peak was fitted by two main components to represent C-C/C-H bond at binding energies about 284.5 eV and C = O bond at higher binding energies about 288 eV. The C = O bond was expected because it is a part of the graphene oxide precursor. Furthermore,during reaction of Ti(SO_4_)_2_ with GO, plenty of oxygen-containing groups, such as C = O located in the interlayer of graphene oxide were consumed during the nucleation and growth of TiO_2_ crystallites, which means graphene oxide has been partly reduced [4]. The oxygen-containing groups on the graphene oxide can interact with titanium hydroxide complex and the TiO_2_ nanoparticles, resulting in hydrogen bonds between them [23].

The ratio between both bonds was almost identical on both samples (about 73%) and had C-C/C-H character; therefore it can be expected that the carbon comes from the precursor rather then from additional contaminations. The other expected bonds as O = C-OH and C-OH were neglected because of the weak signal [24].

The Ti 2p_3/2_ and Ti 2p_1/2_ were identified at binding energies of 458.5 and 464.1 eV, respectively. The position of Ti 2p peaks corresponds to the literature reported values and also to the FWHM values about 1.6 eV for Ti 2p_3/2_ and 2.4 eV for Ti 2p_1/2_, which are reasonable for single peak character. The Ti is probably bonded in the TiO_2_. The equal area/RFS ratios were found on both spin states within precision about 10%. The limited precision is due to the background removal process when the Shirley background was used. The peak positions and the FWHM were found to be practically identical for all investigated samples.

The Additional file 1: Figure S2 shows the IR spectrum of the TiO_2_-GO nanocomposite prepared by thermal hydrolysis of titania peroxo-complexes. The broad absorption peaks about 3427 cm^-1^, and the band at 1631 cm^-1^ correspond to the surface absorbed water and the hydroxyl groups [25]. The band at 1384 cm^-1^ can be assigned to hydroxyl C-OH and that at 1258 cm^-1^ to epoxide C-O-C [26]. The band at 650 cm^-1^ is most probably related to the Ti-O vibration modes of the Ti^3+^-O-Ti^4+^ framework [27].

The Additional file 1: Figure S3 presents the UV–vis absorption spectra of the TiO_2_-GO nanocomposite. The presence of different amounts of GO influences the optical properties of light absorption significantly. With increasing GO content in the nanocomposite light absorption intensity in the UV region grows and a red shift to higher wavelength in the absorption edge at about 400 nm also observed [8]. Increased background in the area 400–800 nm is caused by incorporation of GO into the matrix of TiO_2_[28] and partially also by presence of Ti^3+^.

**Figure 7:**
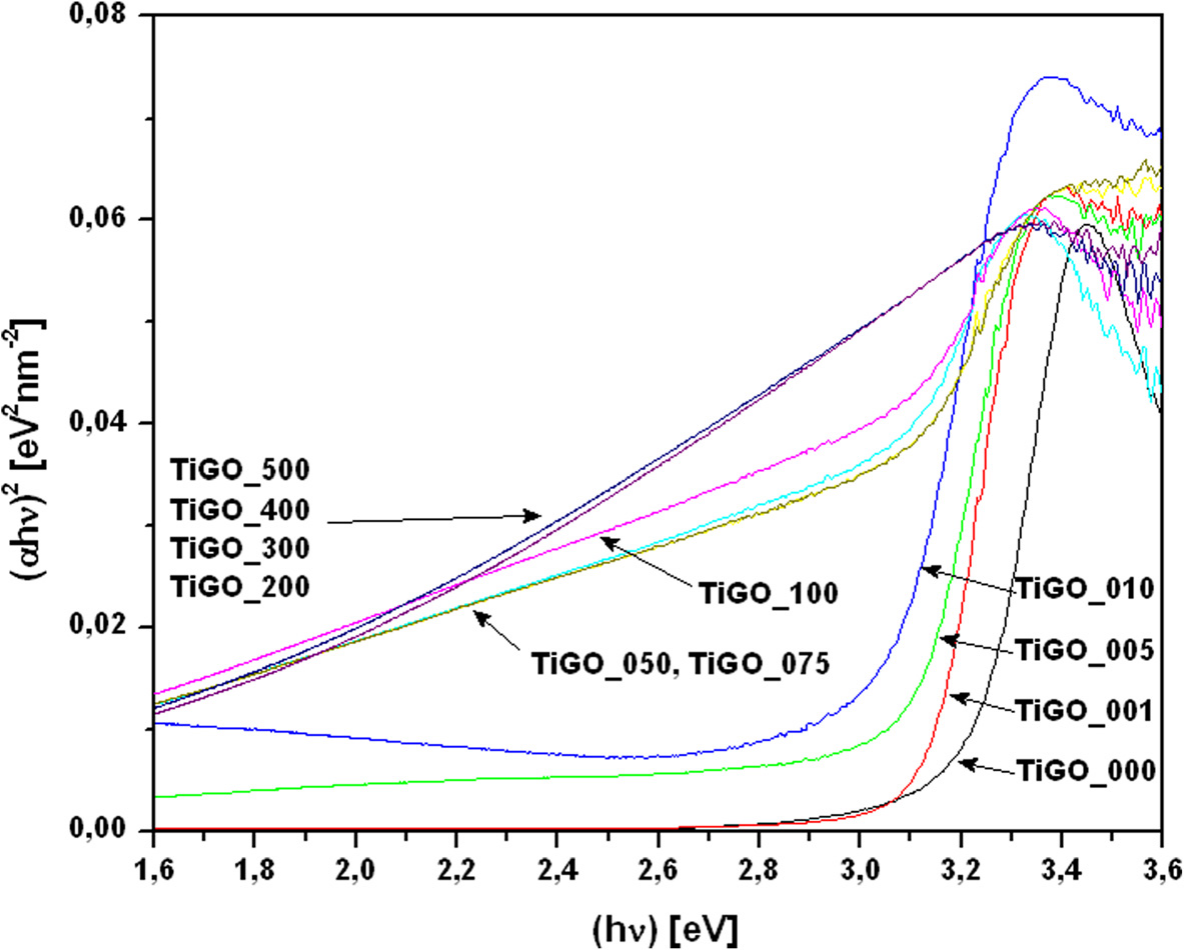
**Band-gap energy (hν) of TiO**_**2**_**-GO composites.**

According to the equation for band gap:

(1) $$\mathrm{\alpha hv}=\text{B}{\left(\text{hv-Ebg}\right)}^{2}$$

where α = (1-R)^2^/2R, R is the reflectance of the "infinitely thick" layer of the solid [29], B is absorption coefficient and hν is the photon energy in eV. Figure 7 plots the relationship of (αhν)^1/2^ versus photon energy (hν = 1239/λ) [30,31], which shows that the band gap of pure TiO_2_ is 3.20 eV, [32] whereas the band gap of the TiO_2_-GO nanocomposite has been reduced from value 3.15 eV (sample TiGO_001) to value <2.50 eV for sample TiGO_100. The band-gaps of samples TiGO_200 to TiGO_500 are impossible to be identified because the absorption edges for TiO_2_ entirely overlapped with those for GO.

The effect of oxidation time variation on the electronic, physical and chemical properties of GO was investigated and it was found that the bandgap of GO increased with oxygen content of the GO material [33]. GO has a bandgap between 1.7 and 4.3 eV in dependence on the preparation method, and it is tunable in the interval 1.9 – 2.6 eV in the range of the visible spectrum as required for efficient photocatalysis.

Graphene oxide is non-stoichiometric material, which retains the lamellar structure of graphite. After treatment in the presence of strong acids and oxidation agents, many oxygen-containing groups such as carboxyl (C − OOH), hydroxyl (C − OH), epoxide (C − O − C) become covalently attached to its layers surfaces. In our experiment GO reacted in the presence of Ti-peroxo-complex, and some functional group such as (− OH, -COOH) on the GO surface were removed. We can can suppose that some unpaired ∏ − electrons bonded with the free electrons on the surface TiO_2_ to form a Ti–O–C structure, which then shifted up the valence band edge and reduce d the band gap [34].

On another hand after the photoactivation of titanium dioxide the electrons can easily transfer to the graphene-nanosheets and photoinduced holes migrate into titania; recombination of e^-^ and h^+^ is strongly reduced, which increases the process yield.

The photocatalytic activity of the as-prepared layered of TiO_2_-GO nanocomposites were assessed from the kinetics of the photocatalytic degradation of butane in the oxygen atmosphere. Photocatalytic oxidation of butane are based on the following overall reactions [35]:

(2) $${2\text{C}}_{4}{\text{H}}_{10}{+13\text{O}}_{2}\to {8\mathrm{CO}}_{2}{+10\text{H}}_{2}\text{O}$$

Before each measurement, the photocatalytic reactor is evacuated, then rinsed with the O_2_ and the measurement is run. If the measured gas chromatogram shows only the oxygen peak at retention time 1.52 min then a gas for the photocatalytic decomposition (in our case the butane) can be injected. After measuring the subsequent chromatogram, peaks for the oxygen and the butane are marked each by value of 1. The value of gases, which are expected to be formed due to photo-activity of the TiO_2_-GO nanocomposite (CO, CO_2_) are assigned each by value 0. By this normalization of input data is performed and the measurement with time period of 2 hours can be started.

In a typical chromatograph of the effluent obtained from butane photocatalytic degradation only oxygen, carbon monoxide, carbon dioxide, water and butane were detected at retention times of 1.52, 1.54, 2.41, 6.42 and 9.53 min [27]. The normalized total ion current (TIC) for CO, CO_2_ and H_2_O after time reaction 60 hours is presented in Table 2.

**Table 2:** **Rate constant k and TIC for CO, CO**_**2 **_**and H**_**2**_**O of TiO**_**2**_**-graphene oxide**

**Sample**	**k 365 nm [h**^**-1**^**]**	**CO [TIC]**	**CO**_**2 **_**[TIC]**	**H**_**2**_**O [TIC]**	**k 400 nm [h**^**-1**^**]**	**CO [TIC]**	**CO**_**2 **_**[TIC]**	**H**_**2**_**O [TIC]**
TiGO_000	0.02686	4.2	7.4	2.1	0.00959	7.2	8.1	2.3
TiGO_001	0.0158	3.7	9.1	0.8	0.00641	16.8	21.1	1.4
TiGO_005	0.01341	9.3	9.1	0.8	0.00451	9.5	12.8	0.8
TiGO_010	0.01356	10.5	11.4	0.7	0.00641	0.6	1.6	0.7
TiGO_050	0.02664	13.1	11.6	1.7	0.00774	1.7	4.7	0.2
TiGO_075	0.00911	8.9	10.6	2.1	0.00774	18.3	18.4	0.7
TiGO_100	0.03012	9.3	7.5	1.7	0.00739	12.4	15.4	0.7
TiGO_200	0.01663	6.7	13.2	1.1	0.00705	13.8	20.4	0.6
TiGO_300	0.00751	19.6	20.5	3.5	0.00517	14.7	19.5	0.8
TiGO_400	0.00683	9.1	9.6	1.9	0.00566	6.6	5.5	1.5
TiGO_500	0.00621	8.2	8.5	0.9	0.00512	6.1	4.9	0.9

**Figure 8:**
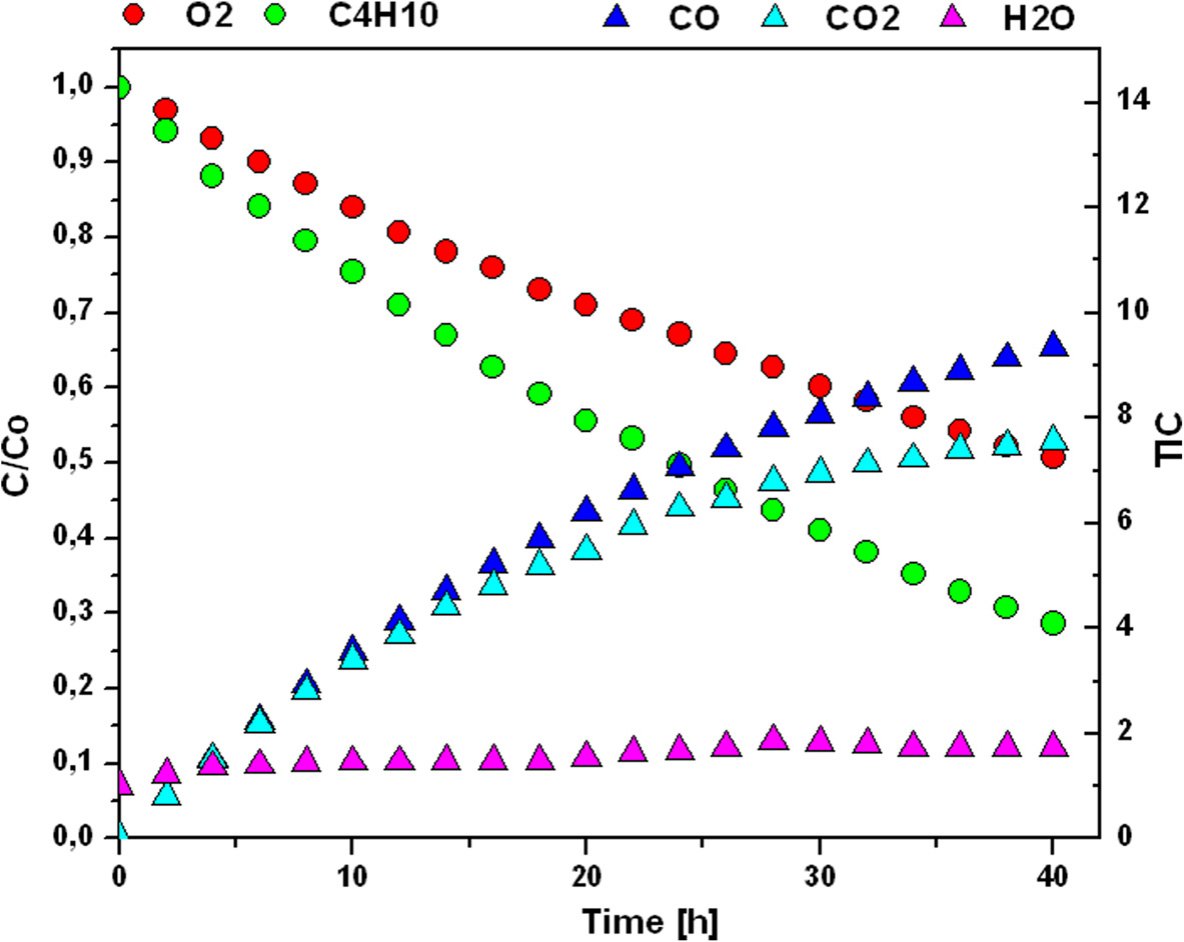
**Typical experimental dependencies of C**_**4**_**H**_**10**_**, O, CO, CO**_**2 **_**and H**_**2**_**O in time.**

The typic corresponding experimental dependencies of butane, oxygen, carbon monoxide and carbon dioxide in time are plotted in Figure 8. The rate of degradation was estimated to obey pseudo-first-order kinetics, and hence the rate constant for degradation k was obtained from the first-order plot according to equation (3),

(3) $$\ln\left(\text{c}/{\text{c}}_{0}\right)=\mathrm{kt}$$

where c_0_ is the initial concentration, c is the concentration of butane after time (t), and k is the first-order rate constant.

The photocatalytic degradation of butane was fitted by curves of the first-order kinetics; the corresponding rate constants are given in Table 2. Djeghri et al. [36] reported photoinduced oxidation of C2–C8 alkanes on TiO_2_ at ambient temperature. In general, they observed that alkanes (C_n_H_2n+2_) formed ketones (C_n_H_2n_O) and other aldehydes C_m_H_2m_O with 2 < m < n. If the alkane was branched, the ketone was C_m_H_2m_O with 3 < m < n. The reactivity of different types of carbon atoms followed the sequence: C_tertiary_ > C_quaternary_ > C_secondary_ > C_primary_. Paper [8] shows, that in our case, the reactivity of butane is different and simpler – no intermediate products such as lower alkane or ketones were detected in the gaseous phase. This means that photocatalytic oxidation proceeds not in the gaseous phase but only on the layer surface directly, causing formation of CO, CO_2_ and H_2_O.

The results presented in Table 2 show, that the best photocatalytic activity under UV and visible light have samples denoted TiGO_100 (k = 0.03012 h^-1^) and TiGO_075 (k = 0.00774 h^-1^), respectively. As for the TiGO_075 sample, there is also the largest increase in CO and CO_2_ content. It is evident from Table 2 that for some samples, there is also an increase in CO and CO_2_ at the expense of the primary decomposition of butane and the value for rate constant decreases. This suggests that in these samples a much deeper mineralization occurs, which leads to the preferential decomposition of intermediates to end products of photocatalytic decomposition, i.e. CO_2_ and H_2_O. This increase in photoactivity for some samples is probably due to reduction of Ti^4+^ to Ti^3+^ and oxidation of free ∏ − bonds during preparation. The interaction of CO_2_ molecules with the excited states of Ti^3+^-O- leads to the formation of CO_2_•¯ anion radicals [37]. The CO_2_•¯ anion radicals readily react with poly(hydroxyethyl metacrylate), which leads to the formation of carbon monoxide due to its strong adsorption on Ti sites of the TiO_2_ surface [8]. The functions of the graphene oxide sheets in the nanocomposite are as follows: i) graphene oxide sheets provide a very good support substrate for the deposition of TiO_2_ nanoparticles, ii) graphene oxide sheets can enhance adsorption ability of the TiO_2_ - graphene oxide nanocomposite, iii) Graphene oxide works as electron acceptor and photosensitizer to efficiently enhance the butane photodecomposition.

## Conclusion

Using graphene oxide sheets as a substrate for TiO_2_, we developed a simple ("one-pot") method to prepare nanocomposite by depositing TiO_2_ on the graphene oxide through liquid phase deposition.

The better photocatalytic properties on the TiO_2_-GO nanocomposite systems irrespective of light sources could be attributed to synergy effects including the increase in specific surface area with graphene oxide amount as well as to the formation of both π–π conjugations between butane molecules and aromatic rings and the ionic interactions between butane and oxygen-containing functional groups at the edges or on the surfaces of carbon-based nanosheets. Graphene oxide works as the adsorbent, electron acceptor and photosensitizer and efficiently enhances the butane photodecomposition. The best photocatalytic activity under UV and visible light was observed for samples denoted TiGO_100 (k = 0.03012 h^-1^) and TiGO_075 (k = 0.00774 h^-1^), respectively. Both the used synthesis route and the GO modification are promising ways to cheap and efficient photocatalysts for the Vis light activation.

## Competing interests

The authors declare that they have no competing interests.

## Authors’ contributions

VŠ was the main author of the work, performed syntheses, and coordinated all characterization and catalytic studies. SB was responsible for electron microscopy, TMG for Raman spectroscopy, MK for XPS measurement, JB assisted with manuscript writing. All authors read and approved the final manuscript.

## Supplementary Material

Additional file 1: Figure S1 The Raman spectrum of prepared graphene oxide. **Figure S2.** IR spectrum of the TiO_2_-GO nanocomposite. **Figure S3.** UV–vis absorption spectra of the TiO_2_-GO. **Table S1.** C Composition of nanocompsite base on XPS. Click here for file

## References

[B1] Krishnamoorthy K Mohan R Kim SJ Graphene oxide as a photocatalytic material Appl Phys Lett 2011 98 24

[B2] Lambert TN Chavez CA Hernandez-Sanchez B Lu P Bell NS Ambrosini A Friedman T Boyle TJ Wheeler DR Huber DL Synthesis and characterization of titania-graphene nanocomposites J Phys Chem C 2009 113 46 19812 19823 10.1021/jp905456f

[B3] Chen C Cai W Long M Zhou B Wu Y Wu D Feng Y Synthesis of visible-light responsive graphene oxide/TiO2 composites with p/n heterojunction ACS Nano 2010 4 11 6425 6432 10.1021/nn102130m 20945928

[B4] Zhang Q He Y Chen X Hu D Li L Yin T Ji L Structure and photocatalytic properties of TiO2-graphene oxide intercalated composite Chinese Sci Bull 2011 56 3 331 339 10.1007/s11434-010-3111-x

[B5] Min S Lu G Dye-sensitized reduced graphene oxide photocatalysts for highly efficient visible-light-driven water reduction J Phys Chem C 2011 115 28 13938 13945 10.1021/jp203750z

[B6] Liang YT Vijayan BK Gray KA Hersam MC Minimizing graphene defects enhances titania nanocomposite-based photocatalytic reduction of CO2 for improved solar fuel production Nano Lett 2011 11 7 2865 2870 10.1021/nl2012906 21688817

[B7] Jiang G Lin Z Chen C Zhu L Chang Q Wang N Wei W Tang H TiO2 nanoparticles assembled on graphene oxide nanosheets with high photocatalytic activity for removal of pollutants Carbon 2011 49 8 2693 2701 10.1016/j.carbon.2011.02.059

[B8] Stengl V Popelkova D Vlacil P TiO2-graphene nanocomposite as high performace photocatalysts J Phys Chem C 2011 115 51 25209 25218 10.1021/jp207515z

[B9] Stengl V Preparation of graphene by using an intense cavitation field in a pressurized ultrasonic reactor Chem - Eur J 2012 10.1002/chem.201201411 23015465

[B10] JCPDS PDF 2 database, Release 50 2000 Newtown Square: International Centre for Diffraction Data

[B11] ICSD ICSD Database 2008 Germany: FIZ Karlsruhe

[B12] Brunauer S Emmett PH Teller E Adsorption of gases in multimolecular layers J Am Chem Soc 1938 60 309 319 10.1021/ja01269a023

[B13] Barrett EP Joyner LG Halenda PP The determination of pore volume and area distributions in porous substances.1. Computations from nitrogen isotherms J Am Chem Soc 1951 73 1 373 380 10.1021/ja01145a126

[B14] Orel ZC Gunde MK Orel B Application of the Kubelka-Munk theory for the determination of the optical properties of solar absorbing paints Prog Org Coat 1997 30 1–2 59 66

[B15] Stengl V Houskova V Bakardjieva S Murafa N Havlin V Optically transparent titanium dioxide particles incorporated in poly(hydroxyethyl methacrylate) thin layers J Phys Chem C 2008 112 50 19979 19985 10.1021/jp803194p

[B16] Stankovich S Dikin DA Piner RD Kohlhaas KA Kleinhammes A Jia Y Wu Y Nguyen ST Ruoff RS Synthesis of graphene-based nanosheets via chemical reduction of exfoliated graphite oxide Carbon 2007 45 7 1558 1565 10.1016/j.carbon.2007.02.034

[B17] Wang GX Yang J Park J Gou XL Wang B Liu H Yao J Facile synthesis and characterization of graphene nanosheets J Phys Chem C 2008 112 22 8192 8195 10.1021/jp710931h

[B18] Jung HG Myung ST Yoon CS Son SB Oh KH Amine K Scrosati B Sun YK Microscale spherical carbon-coated Li4Ti5O12 as ultra high power anode material for lithium batteries Energy Environ Sci 2011 4 4 1345 1351 10.1039/c0ee00620c

[B19] Murafa N Stengl V Houskova V Monodispersed spindle-like particles of titania Microsc Microanal 2009 15 1036 1037 10.1017/S1431927609097359

[B20] Lowell S Shields JE Powder Surface Area and Porosity 1998

[B21] de Boer JA In Structure & Properties of Porous Materials 1958

[B22] Ookubo A Kanezaki E Ooi K ESR, XRD, and DRS studies of paramagnetic Ti3+ ions in a colloidal solid of titanium-oxide prepared by the hydrolysis of TiCl3 Langmuir 1990 6 1 206 209 10.1021/la00091a033

[B23] Seredych M Bandosz TJ Effects of surface features on adsorption of SO2 on graphite oxide/Zr(OH)4 composites J Phys Chem C 2010 114 34 14552 14560 10.1021/jp1051479

[B24] Yang D Velamakanni A Bozoklu G Park S Stoller M Piner RD Stankovich S Jung I Field DA Ventrice CA Jr Ruoff RS Chemical analysis of graphene oxide films after heat and chemical treatments by X-ray photoelectron and Micro-Raman spectroscopy Carbon 2009 47 1 145 152 10.1016/j.carbon.2008.09.045

[B25] Shao GS Zhang XJ Yuan ZY Preparation and photocatalytic activity of hierarchically mesoporous-macroporous TiO2-xNx Appl Catal Environ 2008 82 3–4 208 218

[B26] Lam E Chong JH Majid E Liu Y Hrapovic S Leung ACW Luong JHT Carbocatalytic dehydration of xylose to furfural in water Carbon 2012 50 3 1033 1043 10.1016/j.carbon.2011.10.007

[B27] Bentley FF Smithson LD Rozek AL Infrared Spektra and Characteristic Frequencies 700–300 cm-1 1968 New-York: Wiley-Interscience

[B28] Zhang Y Tang Z-R Fu X Xu Y-J TiO2-graphene nanocomposites for gas-phase photocatalytic degradation of volatile aromatic pollutant: is TiO2-graphene truly different from other TiO2‚àíCarbon composite materials? ACS Nano 2010 4 12 7303 7314 10.1021/nn1024219 21117654

[B29] Christy AA Kvalheim OM Velapoldi RA Quantitative-analysis in diffuse-reflectance spectrometry - a modified Kubelka-Munk equation Vib Spectrosc 1995 9 1 19 27 10.1016/0924-2031(94)00065-O

[B30] Reddy KM Manorama SV Reddy AR Bandgap studies on anatase titanium dioxide nanoparticles Mater Chem Phys 2003 78 1 239 245 10.1016/S0254-0584(02)00343-7

[B31] Tauc J Grigorov R Vancu A Optical properties and electronic structure of amorphous germanium Phys Status Solidi 1966 15 2 627 637 10.1002/pssb.19660150224

[B32] Serpone N Lawless D Khairutdinov R Size effects on the photophysical properties of colloidal anatase TiO2 particles - size quantization or direct transitions in this indirect semiconductor J Phys Chem 1995 99 45 16646 16654 10.1021/j100045a026

[B33] Jeong HK Jin MH So KP Lim SC Lee YH Tailoring the characteristics of graphite oxides by different oxidation times J Phys D: Appl Phys 2009 42 6 Article number 065418

[B34] Zhang Y Pan C TiO2/graphene composite from thermal reaction of graphene oxide and its photocatalytic activity in visible light J Mater Sci 2011 46 8 2622 2626 10.1007/s10853-010-5116-x

[B35] Lorences MJ Patience GS Diez FV Coca J Transient n-butane partial oxidation kinetics over VPO Appl Catal A-General 2004 263 2 193 202 10.1016/j.apcata.2003.12.023

[B36] Djeghri N Formenti M Juillet F Teichner SJ Photointeraction on surface of titanium-dioxide between oxygen and alkanes Faraday Discuss 1974 58 185 193

[B37] Sasirekha N Basha SJS Shanthi K Photocatalytic performance of Ru doped anatase mounted on silica for reduction of carbon dioxide Appl Catal Environ 2006 62 1–2 169 180

