# Supplementary material for: TiO2-graphene oxide nanocomposite as advanced photocatalytic materials
Source: Chem Cent J. 2013 Feb 27;7:41. doi: 10.1186/1752-153X-7-41 (PMC3598647; doi:10.1186/1752-153X-7-41)
Supplement: Additional file 1: Figure S1 — The Raman spectrum of prepared graphene oxide. Figure S2. IR spectrum of the TiO2-GO nanocomposite. Figure S3. UV–vis absorption spectra of the TiO2-GO. Table S1. C Composition of nanocompsite base on XPS. [file 1752-153X-7-41-S1.pdf]

Supplement Information

TiO_2-Graphene Oxide Nanocomposite as Advanced photocatalytic Materials

[1]Václav Štengl*, [1]Snejana Bakardjieva, [1]Tomáš Matys Grygar, [1]Jana Bludská,

[2]Martin Kormunda

stengl@iic.cas.cz

[1]Department of Solid State Chemistry, Institute of Inorganic Chemistry AS CR v.v.i., 250 68 Řež,

Czech Republic

tel.: 420 2 6617 2193 fax.: 420 2 2094 0157

[2]Department of Physics, Faculty of Science, J.E.Purkyně University in Ústí nad Labem, 400 96 Ustí n. L., Czech Republic

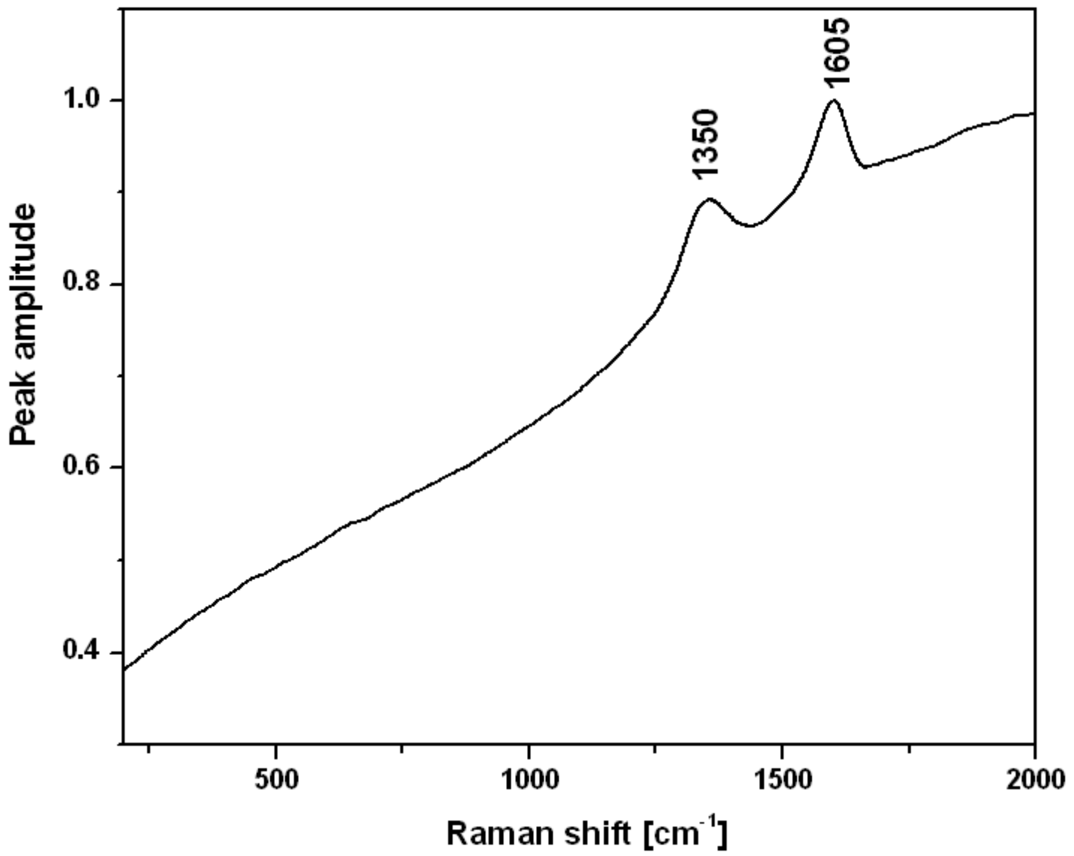

Figure S1. The Raman spectrum of prepared graphene oxide

Table S1. Composition of nanocompsite base on XPS				
Sample	O [at.%]	C [at.%]	Ti [at.%]	O/Ti
TiGO_100	62,00	11,28	26,71	2,32
TiGO_010	66,49	6,68	26,83	248

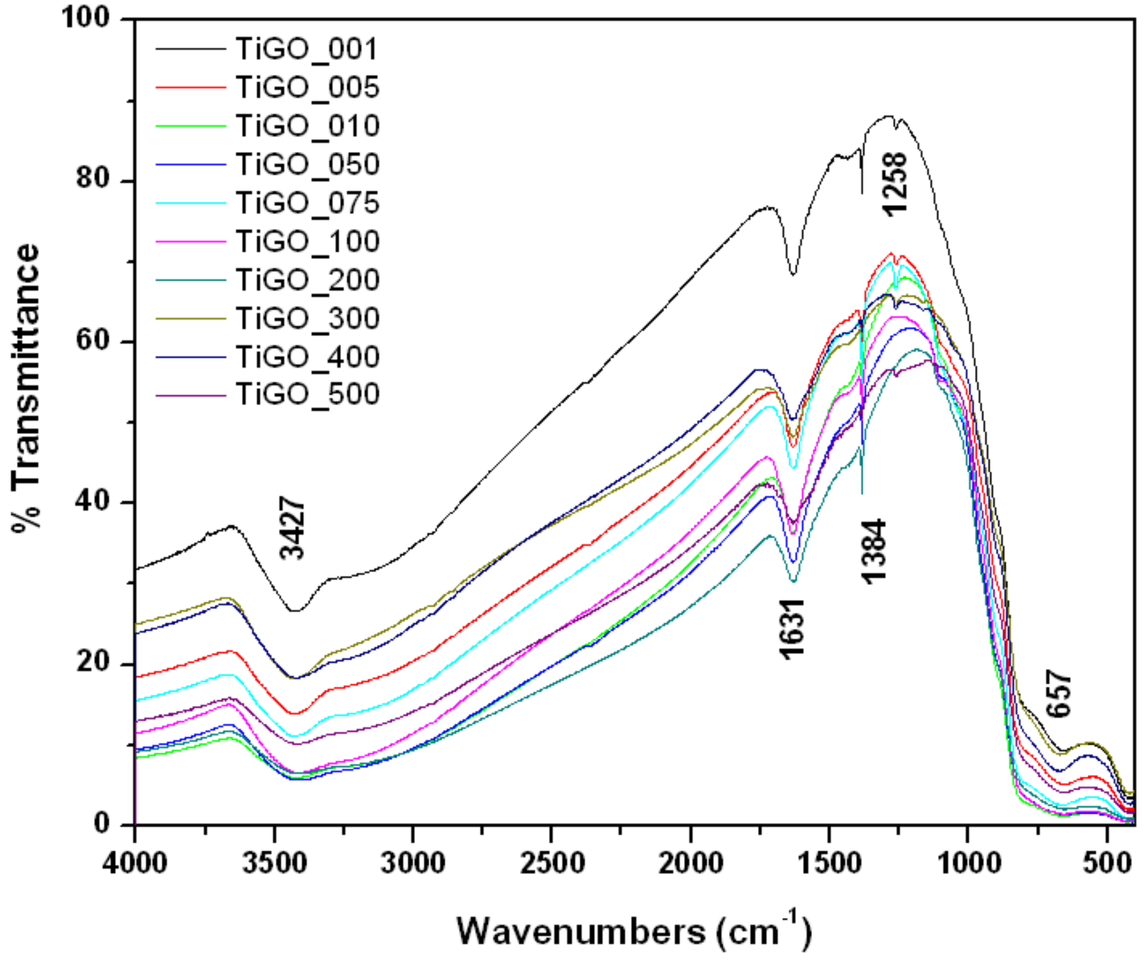

Figure S2. IR spectrum of the TiO_2-GO nanocomposite

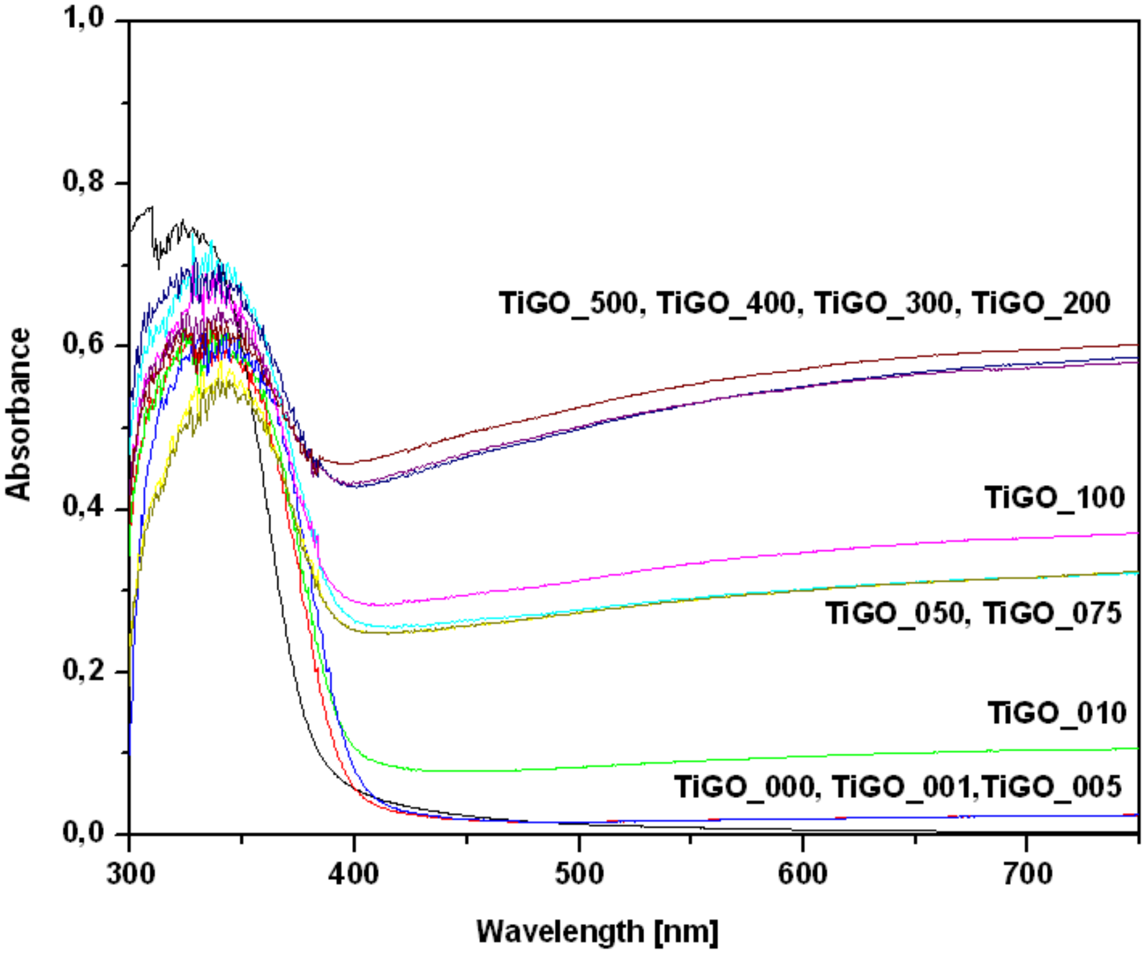

Figure S3. UV-vis absorption spectra of the TiO_2-GO
